# Auxology – an editorial

**DOI:** 10.1186/1824-7288-40-8

**Published:** 2014-01-23

**Authors:** Michael Hermanussen, Barry Bogin

**Affiliations:** 1 Aschauhof 3, 24340, Altenhof, Germany; 2 Centre for Global Health & Human Development, School of Sport, Exercise & Health Sciences, Loughborough University, Loughborough LE11 3TU, UK

**Keywords:** Auxology, Human growth, Body height, Developmental tempo, Community

## Abstract

Auxology (Greek αυξω - I let grow) is the science of human growth and development. Significant public interest focuses on questions like: how does my child grow? How did our ancestors grow? How do other people around the world grow? Are there advantages to being tall and disadvantages to being short? Am I too fat? And many questions are related to the treatment of growth failure.

## 

Ancient Babylonians and Egyptians left us some writings on child growth and variation in height between ethnic groups. In the late 18th century, scattered documents of child growth started to appear in the scientific literature, the studies of Jamberts in 1754 and the annual measurements of the son of Montbeillard published by Buffon in 1777 being the most cited ones [1]. Louis René Villermé (1829) was the first to realize that growth and adult height of an individual depend on the country’s socioeconomic situation. In the 19th century, the number of growth studies rapidly increased, with increasing interest also in growth velocity [2]. Günther documented monthly height increments in a group of 33 boys of various ages [3]. Kotelmann [4] first noted the adolescent growth spurt. In fact, the adolescent growth spurt appears to be a novel achievement in the history of human growth and the amount and intensity of the spurt seems to be greatest in tall and affluent populations [5]. By the beginning of the 20th century, national growth tables were published for most European nations with data for height, weight, and attempts to relate weight and height, though none of these were references in the proper sense of the word as the data were usually derived from small and unrepresentative samples. After the 1930’s X-ray imaging of hand and wrist became popular for determining bone age. Current auxological knowledge is based on the large national studies performed in the 1950’s, 1960’s and the 1970’s, many of them inaugurated by James Tanner [1]. In the late 1970’s a new school of anthropometric history [6] emerged among historians and economists. The main aim of this school was to evaluate secular changes in conscript height during the last 100–200 years and to associate them with socioeconomic changes and political events in the different countries [7]. In the 1980’s and the 1990’s new mathematical approaches have been added of which the LMS method has strongly been recommended for constructing modern growth reference tables [8,9]: M stands for mean, S stands for a scaling parameter, and L stands for the Box-Cox power that is required to transform the skewed data to normality.

Meanwhile, many national and international growth references are based on this technology. And in view of the general idea of growth and adult height being a mirror of nutritional status, health and wealth [10], these techniques have generally been accepted for routine screening programs in Public Health. Anthropometry has also been considered essential for security purposes, for the usability of industrial products, and it has become routine for car and clothing industries, for furniture, housing, and many other aspects of design in the modern environment.

Growth is defined as an increase in size over time. But the rigid metric of physical time is not directly related to the tempo at which an organism develops, matures and ages. Calendar time differs in its meaning in a fast maturing and in a slow maturing organism. Fast maturing children appear tall and “older” than their calendar age suggests, late maturers appear “too young” and often short even though both may later reach the same adult size. Whereas metric scales exist for height, weight and other *amplitude parameters*, there are no continuous scales for maturation and *developmental tempo*. Instead we are used to work with substitutes like the 5-step Tanner scale for staging puberty, and age equivalents for describing bone maturation. Increasing emphasis has recently been put on separating tempo (the pace of development and maturation) and amplitude (the size at a specific state of maturity) [11].

Many of the traditional concepts of growth have recently been questioned in view of this dichotomy. For more than half a century, scattered observations exist on both tempo and amplitude in starvation and illness. Starved populations are not necessarily short populations but they develop at slow pace; well-nourished and economically affluent populations are not necessarily tall. Brundtland et al. [12] published an excellent example that even longstanding starvation does not influence final height. The marked growth impairment in Oslo schoolgirls at the time of the German occupation during World War II was not impairment in amplitude, it was impairment in tempo. The formerly starved cohorts later achieved normal adult height. Similar observations in war- and post-war school children were published in Germany [13]. Tempo impairment has also been observed in chronic illnesses. Aswani and co-workers [14] showed that cystic fibrosis (CF) patients grow poorly at all ages, but eventually achieve normal final height. Also Wiedemann and co-workers [15] stated that in a group of 4,306 CF patients, the initially low height SD scores increased with age, and normal means for height were reached in the adult age group.

Children from a low socioeconomic background are generally shorter than children from an affluent background. People from poor nations tend to be shorter than people from rich nations. Height is associated with household inequality. But when looking closer, the association between growth, final height and economy becomes less evident. Wealthy people were not always tall. Even the wealthiest European merchants of the 19th century were short, by 21st century standards, and produced short offspring. Mean height of the European nations in the mid-19th century ranged between 161 and 165 cm with no evidence that affluent people (except for aristocrats) were taller [16]. It is important to notice that the secular shift in height was a shift in toto. Height variance did not change since the mid-19th century. In other words, the wealthy members of the historic European societies were shorter than modern people from low socioeconomic strata. Height always clusters around the average, today and in the 19th century.

This is also true for migrants. Maya-American children, born to families from Guatemala who migrated to the United States, shift in height by some +11.5 cm compared to Maya children living in Guatemala. The entire range of migrant height, from shortest to tallest, shifts in toto and clusters near the new taller US target [17]. Migrant populations usually assimilate toward the height of their hosts. This was also seen after the German re-unification in 1989: the short people of East Germany caught up in height towards the new target [18].

These and many other observations illustrate the amazing effect of nutrition and health on developmental tempo, and at the same instant, illustrate that neither one of the two factors exhibits similarly marked effect on the amplitude component of growth as seen in people who change their social and economic background like migrants and merging populations. Using a Bayesian approach we re-analysed height data of the Swiss First Zürich Longitudinal Growth Study and found evidence for an important role of social factors in growth. Adolescents adjust their growth rate towards average height of their age mates [19]. We call this the 'community-effect’ factor. We do not know the specific pathway by which this factor operates, however it appears to stimulate growth in the short and suppresses growth in the tall. The community-effect reduces height scattering within the individual’s social habitat: Tall stature communities generate tall people; short stature communities generate short people.

The community effect is only one of several factors which have recently been proposed as novel mechanisms for growth control (such as epigenetic marks or nutrient sensing). The community effect is an 'old’ idea in that it is an extension of the work by Villermé and other 19th century auxologists. The connection between this older and new research strongly underscores the impact of the social environment on child and adolescent growth.

The study of human auxology is as important and exciting as ever before. Evidence of this may be found in our new book *Auxology: Studying Human Growth and Development*[20]. In this book, 56 anthropologists, endocrinologists, mathematicians and colleagues from other disciplines who are involved in the study of human growth, summarized both the traditional concepts of the understanding of human growth and development, and the new, unexpected and challenging questions of modern auxology.

## Competing interest

The authors declare that they have no competing interests.
